# Effect of long-term treatment of Carvacrol on glucose metabolism in Streptozotocin-induced diabetic mice

**DOI:** 10.1186/s12906-020-02937-0

**Published:** 2020-05-11

**Authors:** Yilang Li, Yunpei Mai, Xiaoxia Qiu, Xiaoqing Chen, Conglin Li, Wenchang Yuan, Ning Hou

**Affiliations:** 1 Key Laboratory of Molecular Target & Clinical Pharmacology, School of Pharmaceutical Sciences and the Fifth Affiliated Hospital, Guangzhou Medical University, Guangzhou, 511436 China; 2 KingMed School of Laboratory Medicine, Guangzhou Medical University, Guangzhou, 511436 China

**Keywords:** Carvacrol, Diabetes, Hyperglycemia, Hepatic enzymes

## Abstract

**Background:**

Carvacrol is a food additive with various bioactivities, including reducing the blood glucose level as well as improvement of heart function, in diabetic mice. We explored the antihyperglycemic effect of carvacrol and its effect on the key hepatic enzymes accounting for glucose metabolism.

**Methods:**

A streptozotocin (STZ)-induced diabetes-mellitus model in mice was used. Mice were divided randomly into a control group, diabetic group, low dose carvacrol-treated diabetic group (10 mg/kg body weight [BW]), and high dose carvacrol-treated diabetic group (20 mg/kg BW). Carvacrol was injected intraperitoneally (i.p.) in each carvacrol-treated group daily for 4 weeks and 6 weeks, respectively. The level of random plasma glucose, fasting plasma glucose, and plasma insulin was determined at 4 weeks and 6 weeks after carvacrol administration. The plasma level of total cholesterol (TC), triglycerides (TG), aspartate aminotransferase (AST), alanine aminotransferase (ALT), alkaline phosphatase (ALP), lactate dehydrogenase (LDH), and the activity of hepatic key enzymes related to glucose metabolism were determined.

**Results:**

Carvacrol treatment decreased the levels of random plasma glucose and fasting plasma glucose, significantly in a dose-dependent manner. A significant improvement in glucose tolerance and a significant decrease in the plasma level of TG were observed in carvacrol-treated diabetic mice at a dose of 20 mg/kg BW compared with that in vehicle-treated diabetic mice. There was no significant difference in the plasma level of TC and insulin between vehicle-treated diabetic mice and carvacrol-treated diabetic mice. Carvacrol treatment at a dose of 20 mg/kg BW significantly reduced the plasma level of LDH but not AST, ALT, or ALP, compared with that in the vehicle-treated diabetic group. The activity of hexokinase (HK), 6-phosphofructokinase (PFK), and citrate synthetase (CS) was increased by carvacrol treatment in diabetic mice.

**Conclusions:**

Carvacrol exerted an anti-hyperglycemic effect in STZ-induced diabetic mice. This was achieved through regulating glucose metabolism by increasing the activity of the hepatic enzymes HK, PFK, and CS.

## Background

Diabetes mellitus (DM) is caused by insulin deficiency as shown by insufficient production of insulin (type-1 diabetes mellitus, T1DM) or insulin resistance (type-2 diabetes mellitus, T2DM) and, thus, leads to chronic hyperglycemia. An increased level of glucose in blood is associated with disturbance of carbohydrate metabolism, which is controlled by enzymes [1]. Chronic hyperglycemia and hyperlipidemia activate oxidative stress, which contributes to the development of diabetic complications, such as cardiovascular diseases, kidney injury, and retina injury [2].

The liver is a vital organ that accounts for glucose utilization (30 to 60% of glucose intake) and regulation of the blood glucose level. Glucose homeostasis is maintained by carbohydrate-metabolism pathways such as aerobic oxidation, anaerobic glycolysis, and glycogen synthesis [3]. A set of key enzymes control carbohydrate metabolism. For example, hexokinase (HK) and 6-phosphofructokinase (PFK) participate in anaerobic glycolysis [4, 5]. Citrate synthetase (CS) is a key enzyme involved in aerobic oxidation [6]. The activity of HK and PFK decrease in T1DM [7]. Meanwhile, DM increases lipolysis, resulting in dyslipidemia, which accounts for the development of atherosclerosis in patients suffering from DM [8].

Carvacrol is a natural monoterpene derivative and the main component of most essential oils extracted from aromatic plants, including oregano, musk, thyme, and thymus [9, 10]. Carvacrol is an approved food additive and has extensive biological effects: anti-inflammatory, immunomodulatory, anti-oxidative stress, anti-tumor, anti-bacteria, anti-apoptosis, and neuron protection [11–17]. Besides, carvacrol works as a modulator of ion channels. For example, carvacrol inhibits expression of transient receptor potential melastatin 7 (TRPM7), whereas it activates transient receptor potential channel A1 (TRPA1) and transient receptor potential vanilloid 3 (TRPV3) [18–20]. Inhibition of TRPM7 expression has been shown to protect against high glucose-induced neuron apoptosis, and knockdown of TRPM7 promotes significant insulin secretion in rat insulinoma INS-1 cells [21, 22]. TRPA1 has been shown to have beneficial effects on glucose homeostasis in DM [23]. TRPV3 could be activated by carvacrol to reduce the extracellular matrix and then mediate fibrosis [24]. Recently, carvacrol was shown to exert an antihyperglycemic effect when used in combination with rosiglitazone in high-fat diet (HFD)-induced T2DM mice [25]. In streptozotocin (STZ)-induced diabetic rats, carvacrol has been shown to reduce the blood glucose level and attenuate cognitive deficits [26]. Besides, previously we showed that carvacrol protected against heart injury in T1DM and T2DM [27]. Also, carvacrol relieves DM-induced aortic hypercontractility, partly by virtue of activating phosphoinositide 3-kinase/protein kinase B (PI3K/AKT) signaling [28]. However, the mechanism by which carvacrol reduces blood glucose in DM is not known. We aimed to investigate the anti-hyperglycemic effect of carvacrol and its actions on the key enzymes of glucose metabolism in STZ-induced T1DM.

## Methods

### Animals and establishment of STZ-induced diabetic mice

The protocols for the use and care of animals were approved by the ethics committee of Guangzhou Medical University (Guangzhou, China) (Approval Number: GY2017–040). Handling and treatment of mice were conducted in strict accordance with ARRIVE (Animal Research: Reporting of In Vivo Experiments) guidelines for reporting experiments involving animals.

Adult male C57BL/6 mice (7–8 weeks, 20.0 ± 2.0 g) were used. Animals were purchased from Guangdong Medical Laboratory Animal Center (Guangzhou, China). Mice were housed in separate cages under a controlled environment (12-h day-night cycle, 50–70% humidity, 24 °C) and had free access to food and water. One-week acclimatization was provided to minimize stress.

T1DM was induced by intraperitoneal injection of STZ (Sigma-Aldrich, Saint Louis, MO, USA) using a dose of 45 mg/kg/day for 5 days, as described previously [29, 30]. Age-matched male C57 mice were used as controls and given an equivalent volume of citrate buffer (pH 4.4, 0.1 mol/L) in a parallel manner. Three days after the final injection, blood samples were collected from the caudal vein. The plasma level of glucose was determined using a glucometer (One-Touch™ Ultra Mini® Blood Glucose Monitoring System; Johnson & Johnson, New Brunswick, NJ, USA). Mice with a plasma glucose level > 16.7 mmol/L were defined as having DM.

### Determination of the carvacrol dose

A preliminary experiment was carried out to explore the effective dose of carvacrol on the plasma glucose level in diabetic mice. After 4 weeks of induction of DM, the plasma glucose level in diabetic mice increased persistently and stably. Carvacrol (Sigma-Aldrich) was administered at various doses (10, 20, 40 mg/kg bodyweight [BW]) to eight mice in each group, respectively. Carvacrol was dissolved in 1% dimethyl sulfoxide (DMSO) (Sigma-Aldrich) and given by intraperitoneal injection once a day for 2 weeks. Then, the plasma glucose level was measured using the glucometer.

### Experimental design

Four weeks after DM induction, diabetic mice were divided randomly into three groups of 10. The diabetic group (T1DM) received intraperitoneal injection of vehicle (0.1% DMSO). The low-dose carvacrol-treated diabetic group was treated with carvacrol at 10 mg/kg BW (T1DM + CAR10, i.p.). The high dose carvacrol-treated group was treated with carvacrol at 20 mg/kg BW (T1DM + CAR20, i.p.).

**Fig. 1:**
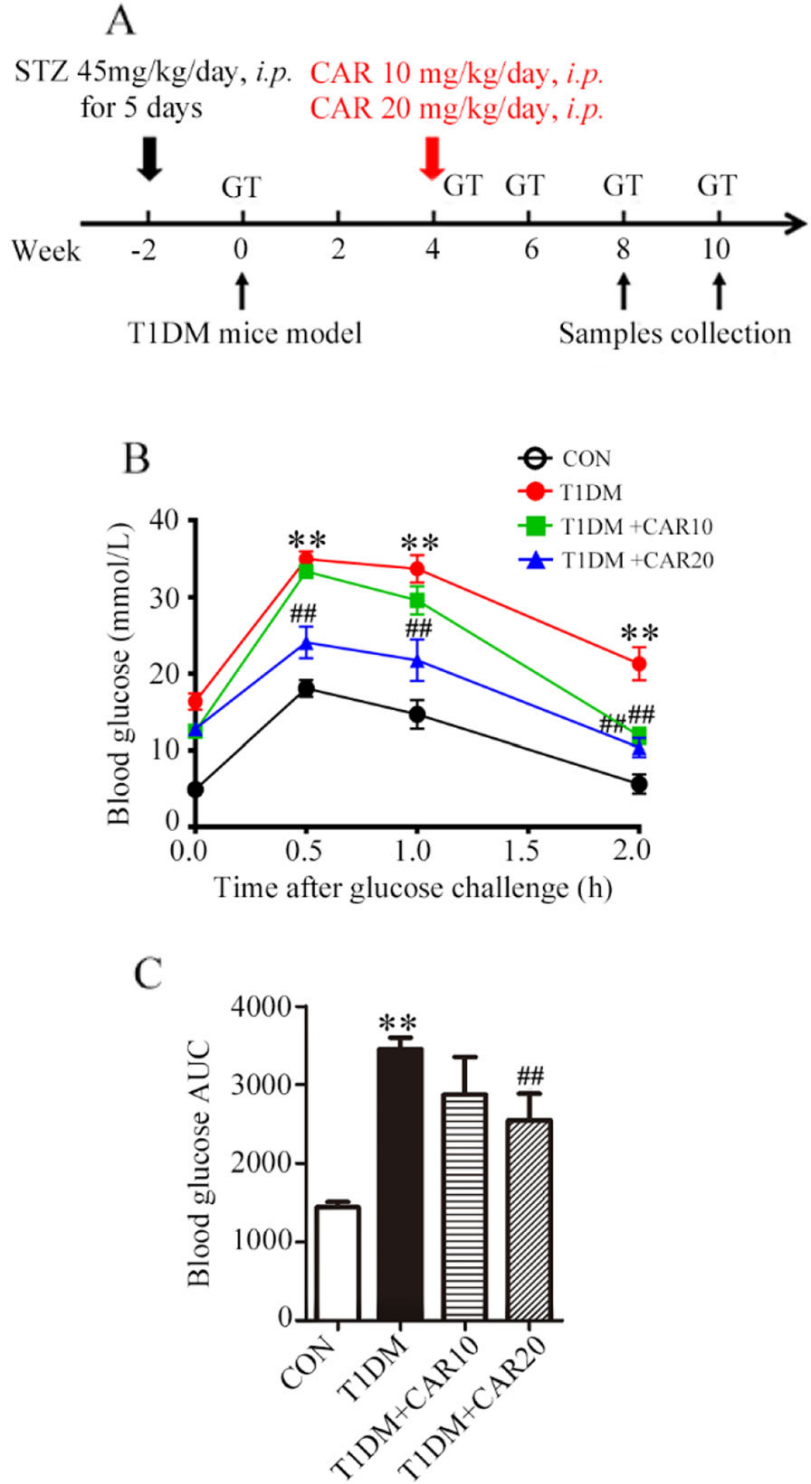
Carvacrol improved glucose tolerance in STZ-induced diabetic mice. **a** Time course of establishment of a T1DM mouse model and carvacrol treatment. STZ was used to establish a T1DM mouse model. After 4 weeks, STZ-induced T1DM mice were treated with two doses of carvacrol (10 mg/kg/day and 20 mg/kg/day) for 4 weeks and 6 weeks. “GT” indicates the glucose level test. **b** and **c** Glucose tolerance test (2 g/kg glucose i.p.) was done in control mice or STZ-induced T1DM mice after 6 weeks of carvacrol treatment or vehicle treatment. **b** The glucose tolerance curve is presented. **c** The mean incremental area under the glucose curve (AUC) was compared between groups. ^**^ versus Con group, ^##^ versus T1DM group, *p* < 0.01, *n* = 10

Carvacrol was prepared fresh and injected via the intraperitoneal route daily for 4 weeks or 6 weeks. Mice in the control group (*n* = 10) received an equivalent volume of 0.1% DMSO given via the intraperitoneal route. During the treatment period, mice were fed standard mice chow and drank water freely (Fig. 1a).

One day before the experiment was terminated, animals were weighed and fasted for 16 h to measure the fasting plasma glucose level. Then, mice were anesthetized with sodium pentobarbital (50 mg/kg, i.p.) and sacrificed by cervical dislocation to collect blood samples and tissues. Blood samples from retro-orbital puncture were aliquoted to two equal parts. One part of the blood was collected into an ethylene diamine tetra acetic acid-coated tube, and the plasma was obtained by centrifuging the blood at 2000×g for 15 min at 4 °C for the estimation of plasma insulin. The other part of the blood was collected in a clean tube and incubated at least 30 min at room temperature, and the serum was obtained by centrifuging the blood at 2000×g for 10 min at room temperature for the estimation of biomedical indicators. Liver were immediately dissected, washed in ice-cold saline to remove blood, and used for the activity assay of hepatic enzymes.

### Determination of the level of random plasma glucose, fasting plasma glucose, plasma insulin and glucose tolerance

The random plasma glucose level and fasting plasma glucose level were measured using the glucometer. Glucose tolerance was determined by intraperitoneal glucose tolerance tests (IPGTTs) in overnight-fasted mice [31, 32]. After intraperitoneal injection of glucose (2 g/kg), the plasma glucose level was measured at 0, 30, 60, 90, and 120 min in blood samples from the caudal vein using the glucometer. The area under the blood glucose curve (AUC) was measured using the trapezoidal rule [33]. The plasma insulin concentration was determined using an enzyme-linked immunosorbent assay kit (Crystal Chem, Downers Grove, IL, USA) following manufacturer instructions.

### Measurement of biochemical indicators

A series of biochemical indicators was determined using diagnostic kits (Dade Behring Holdings, Shanghai, China) with an auto-analyzer (Siemens Healthcare Diagnostics, Munich, Germany). The biochemical indicators were the level of total cholesterol (TC), triglycerides (TG), aspartate aminotransferase (AST), alanine aminotransferase (ALT), alkaline phosphatase (ALP), and lactate dehydrogenase (LDH).

### Measurement of the activity of hepatic enzymes in carbohydrate metabolism

The activity of HK, PFK, and CS in hepatic tissue was determined using commercial quantitative kits (Nanjing Jiancheng Bioengineering Institute, Nanjing, China) according to manufacturer instructions.

### Statistical analyses

Data are the mean ± SEM. The difference in data between groups was analyzed using SPSS v18.0 (IBM, Armonk, NY, USA). One-way ANOVA with Bonferroni post hoc testing was used for comparison of multiple groups. *p* < 0.05 was considered significant.

## Results

### Carvacrol treatment reduced the blood glucose level in STZ-induced diabetic mice

First, we treated diabetic mice with carvacrol at 10, 20, and 40 mg/kg BW per day for 2 weeks. The glucose level increased significantly in diabetic mice compared with that in control mice (*p* < 0.01, *n* = 8) (Table 1). In carvacrol-treatment groups, a significant antihyperglycemic effect was evident from a dose of 20 mg/kg BW (*p* < 0.05, n = 8), and the reduction in the glucose level was not significantly different between groups of carvacrol at a dose at 20 mg/kg BW and 40 mg/kg BW. Therefore, we used carvacrol at a dose of 10 and 20 mg/kg BW for further evaluations.

**Table 1:** Effects of carvacrol treatment for 2 weeks on the random plasma glucose level in STZ-induced T1DM mice

Group	Random plasma glucose (mmol/L)	
	Onset	2 weeks
Con	7.67 ± 0.58	8.24 ± 0.63
TDM	28.26 ± 1.35^**^	30.86 ± 1.01^**^
T1DM + CAR10	27.49 ± 1.51	26.24 ± 1.50
T1DM + CAR20	26.76 ± 1.51	24.74 ± 1.15^#^
T1DM + CAR40	28.76 ± 1.17	24.33 ± 0.76^#^

*Con* Control mice treated with vehicle, *T1DM* STZ-induced T1DM mice treated with vehicle, *T1DM + CAR10* Diabetic mice treated with 10 mg/kg BW/day of carvacrol, *T1DM + CAR20* Diabetic mice treated with 20 mg/kg BW/day/day of carvacrol, *T1DM + CAR40* Diabetic mice treated with 40 mg/kg BW/day of carvacrol. ^**^p < 0.01 versus Con group; ^#^*p* < 0.05 versus T1DM group, *n* = 8

Next, we extended the administration time to 4 weeks and 6 weeks, respectively, to investigate the long-term antihyperglycemic effects of carvacrol in diabetic mice. Carvacrol reduced the level of random plasma glucose in a dose-dependent manner (Table 2). Carvacrol treatment for 4 weeks reduced the level of random plasma glucose to 23.52 ± 1.18 mmol/L at a dose of 10 mg/kg BW (T1DM + CAR10 group) and 21.07 ± 1.23 mmol/L at a dose of 20 mg/kg BW (T1DM + CAR20 group), which were significantly lower than that in the vehicle-treated diabetic group (T1DM group: 27.21 ± 0.93 mmol/L). The level of random plasma glucose was reduced further to 20.70 ± 1.47 mmol/L and 17.08 ± 1.60 mmol/L in diabetic mice treated with carvacrol at a dose of 10 and 20 mg/kg BW for 6 weeks, respectively. Simultaneously, carvacrol treatment also reduced the level of fasting plasma glucose in a dose-dependent manner. Carvacrol treatment at a dose of 20 mg/kg BW decreased the level of fasting plasma glucose by 21.90 and 32.70% of vehicle-treated diabetic mice at 4 weeks and 6 weeks, respectively.

**Table 2:** Effects of carvacrol on the level of random plasma glucose and fasting plasma glucose in STZ-induced diabetic mice

Group	Random plasma glucose (mmol/L)		Fasting plasma glucose (mmol/L)	
	4 weeks	6 weeks	4 weeks	6 weeks
Con	7.81 ± 0.31	7.28 ± 0.94	5.86 ± 0.50	6.07 ± 0.35
T1DM	27.21 ± 0.93**	32.15 ± 1.08**	9.63 ± 0.70**	16.33 ± 0.87**
T1DM + CAR10	23.52 ± 1.18#	20.70 ± 1.47##	8.56 ± 0.48	14.54 ± 1.27
T1DM + CAR20	21.07 ± 1.23##	17.08 ± 1.60##	7.90 ± 0.40#	10.99 ± 0.76##

** *p* < 0.01 versus Con group; # *p* < 0.05, ## *p* < 0.01 versus T1DM group, *n* = 10

### Carvacrol treatment improved glucose tolerance in STZ-induced diabetic mice

We used the IPGTT (the best-established method to determine insulin resistance) to measure glucose tolerance. The blood glucose level increased significantly 30 min after carrying out the IPGTT in vehicle-treated diabetic mice compared with that in control mice (Fig. 1b). Carvacrol treatment at a dose of 10 mg/kg BW reduced the blood glucose level significantly 2 h after the IPGTT (*p* < 0.05, *n* = 10). The higher dose of carvacrol (20 mg/kg BW) exerted more evident effects than the lower dose of carvacrol, showing that treatment reduced the blood glucose level significantly 30 min, 1 h, and 2 h after the IPGTT (*p* < 0.01, *n* = 10). Moreover, The AUC of blood glucose curve increased significantly in vehicle-treated diabetic mice. The AUC of blood glucose curve was reduced significantly by treatment with carvacrol at a dose of 20 mg/kg BW (*p* < 0.01, *n* = 10).

### Effects of carvacrol treatment on the plasma insulin level

The plasma insulin level decreased significantly in vehicle-treated diabetic mice compared with that in control mice (*p* < 0.05, n = 10) (Table 3). Carvacrol treatment at either dose did not change the plasma level of insulin significantly in diabetic mice compared with that in vehicle-treated diabetic mice.

**Table 3:** Effect of carvacrol on the plasma level of insulin in STZ-induced diabetic mice

Group	Insulin (mIU/L)	
	4 weeks	6 weeks
Con	53.9 ± 2.51	55.2 ± 5.80
T1DM	31.65 ± 3.28*	30.00 ± 3.94*
T1DM + CAR10	32.44 ± 3.57	33.02 ± 5.90
T1DM + CAR20	35.14 ± 2.39	37.79 ± 6.69

^*^p < 0.05 versus Con group, *n* = 10

### Effects of carvacrol treatment on the serum level of TC and TG

There was no significant difference in the serum level of TC between the control group, vehicle-treated diabetic group, and carvacrol-treated diabetic group (Table 4). The plasma level of TG in diabetic mice increased significantly compared with that in normal control mice at 4 weeks and 6 weeks (*p* < 0.05, *n* = 10). Carvacrol treatment at a dose of 20 mg/kg BW reduced the level of TG significantly by 33.0 and 43.2% in the diabetic group 4 weeks and 6 weeks after carvacrol treatment, respectively (*p* < 0.05, n = 10). Carvacrol treatment at a dose of 10 mg/kg BW reduced the TG level at 6 weeks, but not 4 weeks, after carvacrol treatment.

**Table 4:** Effects of carvacrol on the plasma level of total cholesterol (TC) and triglycerides (TG) in STZ-induced T1DM mice

Group	TC (mmol/L)		TG (mmol/L)	
	4 weeks	6 weeks	4 weeks	6 weeks
Con	2.06 ± 0.43	2.76 ± 0.51	1.30 ± 0.16	1.37 ± 0.17
T1DM	3.01 ± 0.88	3.25 ± 0.30	2.18 ± 0.18*	2.85 ± 0.24**
T1DM + CAR10	2.98 ± 0.32	2.96 ± 0.59	1.77 ± 0.16	1.91 ± 0.44#
T1DM + CAR20	2.72 ± 0.40	2.59 ± 0.38	1.40 ± 0.13#	1.62 ± 0.19##

^*^p < 0.05, ^**^p < 0.01 versus Con group; ^#^*p* < 0.05, ^##^*p* < 0.01 versus T1DM group, *n* = 10

### Effects of carvacrol treatment on the serum level of AST, ALT, ALP, and LDH

A significant increase in the level of ALT, ALP, and LDH was observed in the diabetic group compared with that in the control group at 4 weeks and 6 weeks (Table 5). The AST level increased significantly in the diabetic group at 6 weeks but not at 4 weeks. Carvacrol treatment had no effect on the serum level of AST, ALT, or ALP at either time point, but reduced the serum LDH level significantly 6 weeks after carvacrol treatment.

**Table 5:** Effects of carvacrol on the plasma level of aspartate aminotransferase (AST), alanine aminotransferase (ALT), alkaline phosphatase (ALP), and lactate dehydrogenase (LDH) in STZ-induced T1DM mice

Group	AST (mmol/L)		ALT (mmol/L)		ALP (g)		LDH (mmol/L)	
	4 weeks	6 weeks	4 weeks	6 weeks	4 weeks	6 weeks	4 weeks	6 weeks
Con	184.2 ± 15.9	240.2 ± 4.4	38.2 ± 4.1	6.07 ± 0.35	28.67 ± 0.53	30.75 ± 1.11	2.06 ± 0.23	2.76 ± 0.21
T1DM	174.3 ± 5.3	351.1 ± 4.9*	50.9 ± 2.2*	16.33 ± 0.87**	23.28 ± 0.30**	22.25 ± 0.70**	3.01 ± 0.08*	3.35 ± 0.10*
T1DM + CAR10	174.8 ± 9.0	365.0 ± 3.5	49.9 ± 4.6	14.54 ± 1.27	21.92 ± 0.33	20.89 ± 0.44	2.78 ± 0.18	2.86 ± 0.09
T1DM + CAR20	188.4 ± 13.1	347.1 ± 6.0	53.7 ± 6.1	12.99 ± 0.76	21.41 ± 0.39	21.00 ± 0.47	2.62 ± 0.10	2.59 ± 0.08#

^*^p < 0.05, ^**^p < 0.01 versus Con group; ^#^*p* < 0.05 versus T1DM group, *n* = 8

### Effects of carvacrol treatment on the activity of hepatic enzymes related to carbohydrate metabolism

**Fig. 2:**
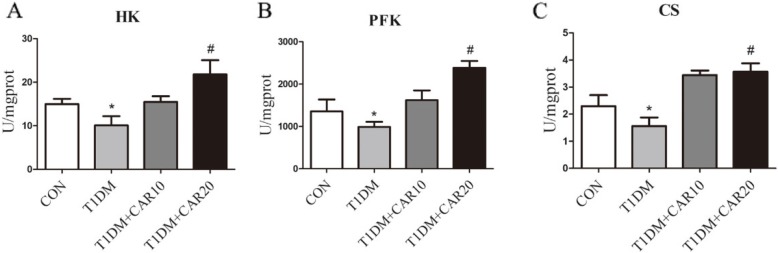
Effect of carvacrol on the activity of glycometabolic enzymes in the liver of STZ-induced T1DM mice. Animals were treated as indicated for 6 weeks, then the enzyme activity was determined using corresponding kits. ^*^p < 0.05 versus Con group; ^#^*p* < 0.05 versus T1DM group, *n* = 10

The activity of HK, PFK and CS in the liver decreased significantly in diabetic mice compared with that in normal control mice (*p* < 0.05, *n* = 10) (Fig. 2). The activity of these hepatic enzymes was reduced significantly by carvacrol treatment at a dose of 20 mg/kg BW.

## Discussion

DM is a metabolic disease characterized by disorders of glycolipid metabolism. Usually, STZ is employed to induce a T1DM model in animals through a mechanism in which STZ destroys pancreatic beta cells selectively. These animals with T1DM display weight loss, insufficient production of insulin, a high glucose level, and hyperlipidemia [34]. Previously, we established a multiple low-dose STZ-induced model of DM in mice [27] that closely resembles T1DM. The established T1DM model in mice was used in the present study.

We found that long-term treatment with carvacrol showed a significant antihyperglycemic effect and reduced the plasma level of TG in STZ-induced diabetic mice. Simultaneously, carvacrol treatment restored the activity of glucose metabolism-related key enzymes (HK, PFK, and CS) significantly and reduced the plasma LDH level as well.

Ezhumalai et al. showed that carvacrol decreased the plasma glucose level in HFD-fed T2DM mice in a dose- and time-dependent manner [25]. In accordance with the present study, our previous study revealed the similar antihyperglycemic effects of carvacrol at a dose of 20 mg/kg BW per day in *db/db* T2DM mice [27]. However, Bayramoglu and co-workers showed that administration of carvacrol at a dose of 25 and 50 mg/kg BW for 7 days did not change the plasma glucose level significantly in STZ-induced T1DM rats [34]. Three main factors might have led to this inconsistent result for carvacrol in these types of diabetic animals. First, DM in T1DM and T2DM mice was induced by a different mechanism. Carvacrol might affect the plasma glucose level through distinct pathways in T1DM and T2DM animals. Second, the preparation of carvacrol between the studies mentioned above was different. The study by Bayramoglu and co-workers in T1DM rats used olive oil to dissolve carvacrol, whereas, in the study by Ezhumalai et al. and in our previous study, carvacrol was dissolved in DMSO. Thus, the availability of carvacrol might have been discrepant between those studies. The third (and most crucial) factor was the different administration period of carvacrol in those studies. Carvacrol was administered for 7 days in T1DM rats, whereas it was given for ≤35 days by Ezhumalai et al. [25] and for 6 weeks in our previous study [27], suggesting that long-term treatment of carvacrol seems to have more apparent effects on the plasma glucose level. This hypothesis was supported by the present study. Our results showed that administration of carvacrol at a dose of 20 mg/kg BW significantly reduced the random plasma glucose level and fasting plasma glucose level in STZ-induced T1DM mice at 4 weeks and 6 weeks. The antihyperglycemic effects of carvacrol were dependent upon its dose. Thus, these results suggest that the hyperglycemia induced by STZ-induced T1DM mice can be alleviated by long-term treatment of carvacrol, thereby demonstrating that carvacrol can reduce the plasma glucose level in T1DM and T2DM. Moreover, our results showed that STZ-induced T1DM mice had impaired glucose tolerance, which is consistent with the work of Tekula and collaborators [35]. Carvacrol treatment for 6 weeks improved glucose tolerance significantly without a change in the plasma insulin level compared with that in vehicle-treated diabetic mice. Our previous study showed that carvacrol treatment increased the level of phosphorylated AKT, which serves as a key component of insulin signaling [27]. These evidences suggest that the antihyperglycemic effects of carvacrol might occur through promotion of insulin sensitivity. Moreover, improvement of insulin resistance is also an essential antihyperglycemic approach for T2DM treatment. Improvement of insulin resistance is probably a common mechanism involved in the antihyperglycemic effects of carvacrol in T1DM and T2DM. The mechanism involved in these effects requires further investigation.

Hyperlipidemia is another characteristic of T1DM and T2DM, and can lead to atherosclerosis and other cardiovascular complications [36]. An excessive amount of fatty acids in plasma promote the production of phospholipids and cholesterol in STZ-induced diabetic animals [37]. Antihyperglycemic treatment that improves insulin resistance can improve the lipid profile [38]. Treatment with carvacrol for 7 days has been shown to reduce the TC level significantly in STZ-induced diabetic mice [34]. Long-term treatment (35 days) with carvacrol has been shown to decrease the level of TC, TG, free fatty acids, and phospholipids in HFD-fed mice [38]. In accordance with those studies, our results showed that long-term treatment (4 weeks or 6 weeks) with carvacrol decreased the plasma TG level significantly and reduced the plasma TC level slightly in STZ-induced diabetic mice. Our previous study demonstrated that carvacrol treatment improved heart function as well as cardiac fibrosis in T1DM and T2DM mice [27], suggesting that the improvement in lipid disorders by long-term treatment with carvacrol protects against the cardiovascular complications of DM. Hyperlipemia is associated with abnormal liver function [39]. A sustained increase in the plasma level of glucose and lipids results in liver dysfunction, as exhibited by an increase in the plasma level of AST, ALT, ALP, and LDH [40]. We also observed a significant increase in the level of these liver enzymes in STZ-induced diabetic mice, and carvacrol treatment reduced the LDH level significantly. Ezhumalai et al. showed that carvacrol treatment reduced the plasma level of ALT, AST, and ALP in HFD-induced T2DM mice [25]. Taken together, these results demonstrate that carvacrol treatment attenuates hepatic dysfunction in DM, and provides beneficial effects in DM treatment.

The mechanisms by which carvacrol reduces the plasma glucose level in DM remain largely unclear. Carvacrol can activate TRPA1 and TRPV3 channels, whereas it blocks TRPM7 channels [18–20]. Activation of TRPA1 expression stimulates glucagon-like peptide-1 (GLP-1) secretion [41]. GLP-1 decreases the plasma glucose level by enhancing release of glucose-dependent insulin, inhibiting gastric emptying and the level of postprandial glucagon, and reducing food intake. Thus, GLP-1 activators are used for T2DM treatment [42]. Therefore, the antihyperglycemic effects of carvacrol might be through TRPA1-mediated GLP-1 secretion. Besides, carvacrol can activate peroxisome proliferator-activated receptor (PPAR)-γ which works as an “insulin sensitizer” [43]. The PPAR-γ signaling pathway might also be involved in the antihyperglycemic effects of carvacrol.

The liver is responsible for utilization of 30 to 60% of ingested glucose. Glycolysis is an initial process of glucose utilization to produce energy for cellular metabolism. HK is a key enzyme in the first step of anaerobic glycolysis to catalyze glucose to produce glucose 6-phosphate glucose (G-6-P) [4]. G-6-P is the reactive form of glucose, and affects the overall efficiency of glucose metabolism. Then, G-6-P is converted into fructose-6-phosphate followed by catalysis into fructose 1,6-diphosphate by another key enzyme: PFK [3]. Finally, anaerobic glycolysis of glucose generates the product of pyruvate. If oxygen is available, pyruvate serves as the substrate for pyruvate oxidation. Then, the product of pyruvate oxidation, acetyl CoA, enters the tricarboxylic acid cycle and is catalyzed by a key enzyme: CS [11]. It has been reported that the activity of these key enzymes, HK and PFK, decreases in STZ-induced T1DM animals and HFD-induced T2DM animals [4, 5, 25]. In accordance with those studies, we showed that the activity of HK and PFK in the liver decreased significantly in diabetic mice compared with that in normal control mice. We also observed a decrease in CS activity in diabetic mice. Carvacrol treatment increased the activity of these three hepatic key enzymes significantly in diabetic mice. These results suggest that carvacrol treatment strengthens the anaerobic glycolysis pathway involved in glucose metabolism in STZ-induced DM.

## Conclusions

Long-term treatment with carvacrol exerts an antihyperglycemic effect through improving glucose metabolism in STZ-induced T1DM mice. Regulation of glucose metabolism by carvacrol is involved in the anaerobic glycolysis pathway, which is mediated by the hepatic enzymes HK, PFK, and CS. Our study suggests that carvacrol might serve as a drug-development target for DM treatment.

## Data Availability

All data generated or analyzed during this study are included in this published article [and its supplementary information files].

## Abbreviations

ALP: Alkaline phosphatase
ALT: Alanine aminotransferase
AST: Aspartate aminotransferase
ARRIVE: Animal Research: Reporting of In Vivo Experiments
AUC: Area under the curve
BW: Body weight
CS: Citrate synthetase
DM: Diabetes mellitus
DMSO: Dimethyl sulfoxide
GLP-1: Glucagon-like peptide-1
G-6-P: Glucose 6-phosphate glucose
HK: Hexokinase
HFD: High-fat diet
IPGTT: Intraperitoneal glucose tolerance test
LDH: Lactate dehydrogenase
PFK: 6-phosphofructokinase
PI3K/AKT: Phosphoinositide 3-kinase/protein kinase B
PPAR-γ: Peroxisome proliferator-activated receptor
STZ: Streptozotocin
T1DM: Type-1 diabetes mellitus
T2DM: Type-2 diabetes mellitus
TC: Total cholesterol
TG: Triglycerides
TRPA1: Transient receptor potential channel A1
TRPM7: Transient receptor potential melastatin 7
TRPV3: Transient receptor potential vanilloid 3
